# A quality assessment of genetic association studies supporting susceptibility and outcome in acute lung injury

**DOI:** 10.1186/cc7098

**Published:** 2008-10-25

**Authors:** Carlos Flores, Maria del Mar Pino-Yanes, Jesús Villar

**Affiliations:** 1 CIBER de Enfermedades Respiratorias (Instituto de Salud Carlos III), Carretera Soller Km. 12, 07110 Mallorca, Spain; 2 Research Unit, Hospital Universitario NS de Candelaria, Carretera del Rosario s/n, 38010 Santa Cruz de Tenerife, Spain; 3 Multidisciplinary Organ Dysfunction Evaluation Research Network, Research Unit, Hospital Universitario Dr. Negrin, Barranco de la Ballena s/n, 35010 Las Palmas de Gran Canaria, Spain; 4 Keenan Research Center, St. Michael's Hospital, 30 Bond Street, Toronto, ON M5B 1W8, Canada

## Abstract

**Introduction:**

Clinical observations and animal models provide evidence that the development of acute lung injury (ALI), a phenomenon of acute diffuse lung inflammation in critically ill patients, is influenced by genetic factors. Association studies are the main tool for exploring common genetic variations underlying ALI susceptibility and/or outcome. We aimed to assess the quality of positive genetic association studies with ALI susceptibility and/or outcome in adults in order to highlight their consistency and major limitations.

**Methods:**

We conducted a broad PubMed literature search from 1996 to June 2008 for original articles in English supporting a positive association (*P *≤ 0.05) of genetic variants contributing to all-cause ALI susceptibility and/or outcome. Studies were evaluated based on current recommendations using a 10-point quality scoring system derived from 14 criteria, and the gene was considered as the unit of replication. Genes were also categorized according to biological processes using the Gene Ontology.

**Results:**

Our search identified a total of 29 studies reporting positive findings for 16 genes involved mainly in the response to external stimulus and cell signal transduction. The genes encoding for interleukin-6, mannose-binding lectin, surfactant protein B, and angiotensin-converting enzyme were the most replicated across the studies. On average, the studies had an intermediate quality score (median of 4.62 and interquartile range of 3.33 to 6.15).

**Conclusions:**

Although the quality of association studies seems to have improved over the years, more and better designed studies, including the replication of previous findings, with larger sample sizes extended to population groups other than those of European descent, are needed for identifying firm genetic modifiers of ALI.

## Introduction

Critical illness in adults often is followed by acute lung injury (ALI). ALI and its most severe form, the acute respiratory distress syndrome (ARDS), are currently defined as a phenomenon of acute diffuse lung inflammation pathologically characterized by an acute onset of non-cardiogenic pulmonary edema resulting from increased capillary-alveolar permeability. Both are clinically manifested by hypoxemia under mechanical ventilation (arterial partial pressure of oxygen/fraction of inspired oxygen [PaO_2_/FiO_2_] of less than or equal to 300 mm Hg for ALI and PaO_2_/FiO_2 _of less than or equal to 200 mm Hg for ARDS), diffuse bilateral pulmonary infiltrates on chest radiographs, and reduced lung compliance [1]. Pneumonia and sepsis are the main and most common risk conditions associated with the development of both disorders [2]. ALI and ARDS remain a major health problem worldwide: it has been estimated that each year in the US there are 190,600 cases of ALI, which are associated with 74,500 deaths and 3.6 million hospital days [3]. Our understanding of the pathogenesis of ALI and ARDS has improved in recent years with the appreciation that inflammation is a fundamental component of the pathophysiology of these two clinical manifestations of the same syndrome.

Clinicians have long recognized that all critically ill patients with ALI are not alike. It is becoming apparent that the diversity of clinical manifestations and the response to treatment and outcome among patients with the same disease process are influenced by genetic factors [4-6]. The first piece of evidence supporting a role for genetic differences in infection risk and outcome came from an epidemiological study reporting a strong association between death from infection in adoptees and their biological, but not adoptive, parents [7]. For ALI, this is further strengthened by the mortality rate disparities across the different ethnic groups in the US [8]. In addition, ALI models in inbred rodents have demonstrated differences for susceptibility and severity traits, allowing the identification of several loci and pinpointing the multigenic nature of the condition [9-11]. In our attempt to better define patients at risk, recent trends have turned our attention to the search for common genetic variation underlying ALI susceptibility and/or outcome. Based on the extensive evidence that common genetic variation with modest effects underlies susceptibility to common complex diseases [12] and on the impossibility of linkage analysis to detect such signals [13], association studies have constituted the main tool for improving our understanding of the genetic factors affecting ALI susceptibility and outcome.

Association studies compare two groups of samples (cases and controls) for statistical differences in the frequency of variants at one or more sites of the genome. Although the International HapMap Project and the development of genotyping technologies have made possible the testing of more than one million of these variants in a single experiment [14], they have been available for a short period of time [15]. Thus, currently, association studies in ALI have exclusively used a candidate gene approach, in which one or several genes – known to be etiologically involved in the disease – are studied for relevant variant sites. In general, the inconsistency of findings across association studies [16] – partially attributed to inappropriate designs, implementations, and/or interpretations of studies – has motivated the formulation of standards to improve their quality and to help perform meta-analysis [17] under the premise that the replication of previous findings most likely reflects interesting biological processes rather than methodological quirks. Here, we aimed to examine studies reporting positive findings with all-cause ALI susceptibility and/or outcome in adults in order to evaluate their relative merits and caveats based on actual recommendations.

## Materials and methods

### Literature search of genetic association studies

We conducted a broad PubMed literature search from 1996 to June 2008 for original articles by querying for 'polymorphism and acute lung injury' and 'polymorphism and ARDS'. The retrieved references were then manually curated, and those reporting genetic association studies and published in English were sought. Studies were considered if a positive association (*P *≤ 0.05) was reported with either susceptibility or outcomes of all-cause ALI or ARDS. Since the current tendency to perform association analysis at the individual variant level may be problematic (for example, there may be important differences in allele frequency or linkage disequilibrium [LD] structure across different populations), we instead considered the gene as the unit of replication [18]. The Gene Ontology was used to categorize associated genes according to biological processes [19].

### Quality assessment

Among reports with positive associations, study quality – rather than significance value – was reviewed based on current recommendations. Since performing a checklist of all issues to consider in association studies would require more than a single article, we have focused on the most relevant criteria from a checklist suggested recently [20]. All together, 14 criteria were considered and each of them was scored as 1 if present or 0 if absent. Scoring was performed independently by two authors. Studies were divided into case-control or cohort studies based on the design in which the authors reported the positive association. If a case-control study reported a positive association with an outcome in the case series, the positive finding of the study was also considered as found in a cohort design. A final quality score was obtained by adding up scores from all criteria (see below). A reported association could have a maximum score of 14 points for case-control studies if more than one polymorphism was analyzed, a maximum of 13 points if reporting a case-control study for a single polymorphism (multiple testing adjustment not needed) or for a cohort with more than one polymorphism analyzed (definition of the control group not needed), or a maximum of 12 points for cohorts analyzing a single locus (definition of the control group and the multiple testing adjustment are not needed). To facilitate comparison across study designs, scores were then transformed to a 0- to 10-point scale.

Criteria that were evaluated in relation to the study design included power calculation, characterization of cases and controls or the cohort, and whether the study considered common gene-wide variation. Power calculation was scored as present only if it was explored prospectively or retrospectively as part of the original study. Controls were considered to be adequate if obtained from the same population as cases and described in such a way that could be replicated. This criterion was not scored in the cohort studies. Adequacy of case groups was considered if demographical and clinical data were reported in sufficient detail in the text and/or a table. Mentioning accepted international guidelines for phenotype definition [1] as the sole description of cases was not considered to be acceptable. To cover the adequacy of exploring gene-wide variation in the association, LD must have been explored for polymorphism selection and/or for the interpretation of results.

To evaluate study reproducibility, unambiguous identification of polymorphisms by means of National Center for Biotechnology Information (NCBI) reference numbers or flanking sequences was scored as present. The sole description of amplification primer pairs and/or a reference to a previous publication that reported the assay was not considered to be acceptable. The three other criteria evaluated as part of study reproducibility relate to genotyping quality control measures. Duplicate genotyping of a portion of individuals by means of the same or alternative genotyping techniques to calculate an error rate was considered to be adequate and scored as present. Testing of Hardy-Weinberg equilibrium was scored as present even when significant *P *values were reported for any of the groups as long as a duplicate genotyping was performed. Finally, adequate studies performed an interpretation of results blind to the clinical status of samples.

To evaluate the statistical analyses, we considered the presence of multiple testing adjustments to be adequate. However, note that this category was not scored if a single polymorphism was assessed since we did not consider an adjustment for the multiple explored phenotypes or outcomes for the adequacy of the study to be necessary. Three other categories scored as adequate included an evaluation of other recorded risk factors by means of regression models, reporting major findings in terms of risks (as hazard or odds ratios) and their 95% confidence intervals (CIs), and an empirical assessment or adjustment for population stratification by means of an independent set of polymorphic markers.

Finally, we scored as adequate additional support from studies performing a validation in at least a second independent sample as part of the original study. Studies designed to confirm previously associated polymorphisms were not considered to be acceptable for this category. Studies that also included experiments providing evidence of functionality for associated variant(s) were scored as adequate. The sole reference to previous publication(s) providing the functional evidence of the associated polymorphism was scored as absent.

## Results

**Figure 1:**
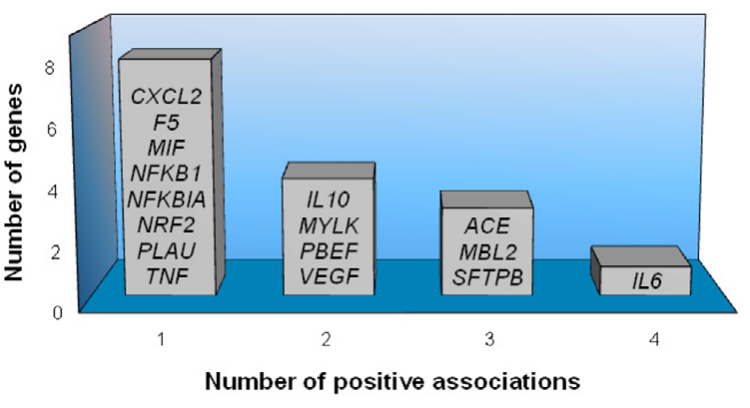
Genes that showed positive association with either susceptibility and/or outcome with all-cause acute lung injury or acute respiratory distress syndrome. ACE, angiotensin-converting enzyme; CXCL2, chemokine CXC motif ligand 2; F5, coagulation factor V; IL-6, interleukin-6; IL-10, interleukin-10; MBL2, mannose-binding lectin-2; MIF, macrophage migration inhibitory factor; MYLK, myosin light-chain kinase; NFKB1, nuclear factor kappa light polypeptide gene enhancer in B cells; NFKBIA, nuclear factor kappa light polypeptide gene enhancer in B cells inhibitor alpha; NRF2, nuclear factor erythroid-derived 2 factor; PBEF, pre-B cell-enhancing factor; PLAU, plasminogen activator urokinase; SFTPB, surfactant pulmonary-associated protein B; TNF, tumor necrosis factor; VEGF, vascular endothelial growth factor.

Searching for 'polymorphism and acute lung injury' or 'polymorphism and ARDS', we retrieved 53 and 23 original articles, respectively. This allowed us to identify a total of 29 articles [21-49] on 16 genes that showed a positive association with susceptibility and/or outcomes of all-cause ALI or ARDS in at least one study (Table 1). Although we used broad terms for this search, the possibility for missing additional studies with positive findings might still exist. Nevertheless, a complementary search querying for the disease name in the HuGeNet Navigator [50] gave completely overlapping results, showing studies for additional genes, albeit reporting negative findings. Most studies (72.3%) were carried out exclusively in populations of European descent (defined as 'Whites' or Caucasians). A minority of studies were performed in East Asians (7%) and the remaining 20.7% of studies included populations of both European and African descent. Among the 16 genes that showed a positive association in at least one study, four genes were replicated in at least a second article, three genes were replicated in at least three studies, and one gene was replicated in four studies (Figure 1). Since with only two exceptions [32,35] none of these studies attempted to validate the association results in an independent sample, all studies were counted as a single contribution for the purpose of this assessment. Ontology analysis of these genes showed that the majority of them were involved in the response to external stimulus (56.2%) and cellular signal transduction (50%). There was also a prominent representation of genes implicated in cell proliferation (43.8%), inflammatory response (37.5%), immune response (25%), and chemotaxis (25%).

**Table 1:** Positive genetic association studies with acute lung injury/acute respiratory distress syndrome susceptibility and/or outcome (by year of publication)

Gene	Associated variant(s)^a^	Sample size (case/control)	Sample size (cohort)	Phenotype(s)	Population	Reference (year)
*SFTPB*	T/C +1580	52:46		ARDS	European	[21] (2000)

*IL-6*	G/C -174		96 ARDS	Mortality	European	[22] (2002)

*ACE*	I/D intron 16	96:2,168		ARDS, mortality	European	[23] (2002)

*SFTPB*	T/C +1580		402 CAP	ARDS	Multiethnic	[24] (2004)

*SFTPB*	Intron 4 TR		189 at risk of ARDS	ARDS	Multiethnic	[25] (2004)

*PBEF*	T/G -1001 and haplotype	87:84		ALI	European	[26] (2005)

*MBL2*	Haplotypes	569:1,188		SARS	Chinese	[27] (2005)

*IL-6*	Gene-wide haplotypes		228 SIRS	ALI, need of MV	European	[28] (2005)

*IL-6*	Haplotype	98:84		ALI	European	[29] (2005)

*TNF*	G/A -308	212:441		ARDS, mortality	European	[30] (2005)

*VEGF*	C/T +936	117:240		ARDS, severity	European	[31] (2005)

*MYLK*	Multiple SNPs and haplotypes	138:146		ALI	Multiethnic	[32] (2006)

*IL-10*	A/G -1082	211:429		ARDS, severity	European	[33] (2006)

*ACE*	I/D intron 16	101:348		ARDS mortality	Chinese	[34] (2006)

*MIF*	Haplotypes	151:173		ALI	Multiethnic	[35] (2007)

*PBEF*	T/G -1001 and haplotype	375:787		ARDS, mortality	European	[36] (2007)

*NRF2*	C/A -617		90 major trauma	ALI	Multiethnic	[37] (2007)

*CXCL2*	-665 TR		183 severe sepsis	ARDS mortality	European	[38] (2007)

*MBL2*	Gly54Asp	212:442		ARDS, severity	European	[39] (2007)

*ACE*	I/D intron 16	84:200		ARDS mortality	European	[40] (2007)

*NFKBIA*	Haplotype	382:828		ARDS	European	[41] (2007)

*NFKB1*	Ins/del ATTG -94		103 ARDS	Severity	European	[42] (2007)

*VEGF*	C/T +936 and haplotype		394 ARDS	Mortality	European	[43] (2007)

*PLAU*	Gene-wide haplotype	175:252		ALI, severity	European	[44] (2008)

*MYLK*	3 variants and multiple haplotypes		273 major trauma	ALI	Multiethnic	[45] (2008)

*MBL2*	Haplotypes		848 CAP	ARDS	European	[46] (2008)

*IL-6*	Gene-wide haplotype	67:96		ALI	European	[47] (2008)

*IL-10*	A/G -1082		100 severe multiple trauma	ARDS	European	[48] (2008)

*F5*	Arg506Gln		106 ARDS	Mortality	European	[49] (2008)

^a^Names are those originally reported in the corresponding reference. Ins/del, insertion-deletion polymorphism. ACE, angiotensin-converting enzyme; ALI, acute lung injury; ARDS, acute respiratory distress syndrome; CAP, community-acquired pneumonia; CXCL2, chemokine CXC motif ligand 2; F5, coagulation factor V; IL-6, interleukin-6; IL-10, interleukin-10; MBL2, mannose-binding lectin-2; MIF, macrophage migration inhibitory factor; MV, mechanical ventilation; MYLK, myosin light-chain kinase; NFKB1, nuclear factor kappa light polypeptide gene enhancer in B cells; NFKBIA, nuclear factor kappa light polypeptide gene enhancer in B cells inhibitor alpha; NRF2, nuclear factor erythroid-derived 2 factor; PBEF, pre-B cell-enhancing factor; PLAU, plasminogen activator urokinase; SARS, severe acute respiratory syndrome; SFTPB, surfactant pulmonary-associated protein B; SIRS, systemic inflammatory response syndrome; SNP, single-nucleotide polymorphism; TNF, tumor necrosis factor; TR, tandem repeat (polymorphism); VEGF, vascular endothelial growth factor.

Seventeen studies (58.6%) reported positive findings using a case-control design and 12 (41.4%) using a cohort. Median sample sizes among studies were of 100 cases (interquartile range [IQR]: 85 to 212) and 200 controls (IQR: 88 to 519), whereas the median sample size for cohort studies was 183 patients (IQR: 100 to 273). Overall median quality score was 4.62 (IQR: 3.33 to 6.15) and maximum and minimum scores were 7.14 and 0.71, respectively. When studies were classified by design, the median quality score in case-controlled studies (5.38; IQR: 4.29 to 6.43) was significantly higher than in cohort studies (3.33; IQR: 2.88 to 5) (*P *= 0.030, Mann-Whitney *U *test). When studies were explored by the year of publication, there was an improvement trend of association studies over time (Spearman rho = 0.38, *P *= 0.041), but this was due mostly to case-controlled studies (Spearman rho = 0.70, *P *= 0.002) since no significant trend was observed for cohort studies (Spearman rho = 0.27, *P *= 0.40).

**Figure 2:**
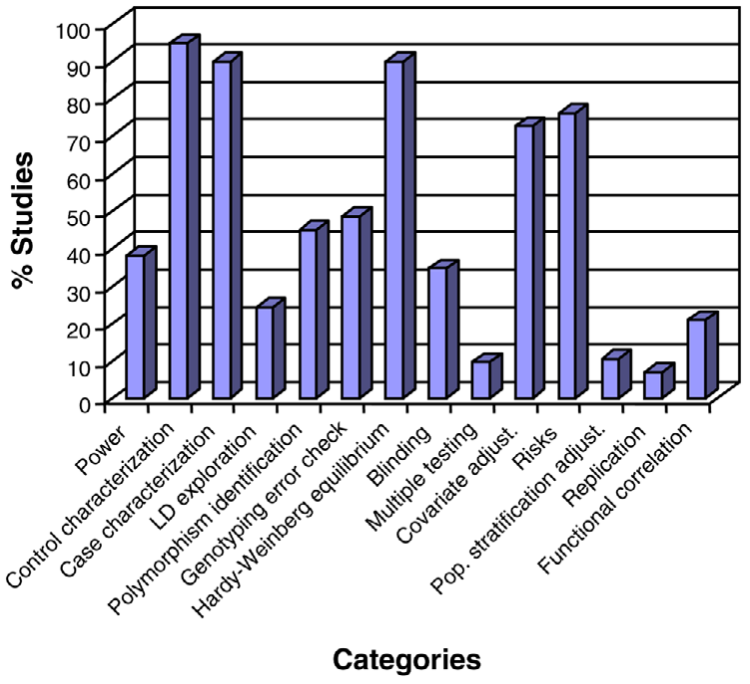
Percentage of studies scored as adequate for 14 criteria (x-axis) used for the quality assessment of genetic association studies supporting susceptibility and/or outcome in acute lung injury. LD, linkage disequilibrium; pop. stratification adjust., population stratification adjustment.

Almost two thirds of the studies (62.1%) did not explore their power to detect positive findings. Nearly all studies (97%) fulfilled the internationally accepted definition criteria for ALI and ARDS [1], and most studies (89.7%) appropriately described demographical and clinical data from cases (Figure 2). More heterogeneity was found for the criteria to select a control group: although most studies used healthy subjects or population-based controls (43%), a great proportion of studies preferred ICU patients as controls (38%). In any case, 94.4% of studies fulfilled the required criteria to have an adequate control group. Most studies (75.9%) analyzed a few variants per gene (34.5% analyzed a single variant with anticipated functionality) without providing appropriate coverage or discussion to other untyped common variation by means of LD-based methods.

In almost half of the studies (44.8%), we were not able to identify the associated polymorphism(s) in NCBI databases straightforwardly and unambiguously since flanking sequences or genetic reference numbers were lacking. Less than half of the studies reported genotyping error checks (48.3%) or a blinding strategy (34.5%) to avoid biased results (Figure 2). However, Hardy-Weinberg equilibrium was assessed separately in cases and controls or in the cohort in 89.7% of studies. Remarkably, three of these studies reported a positive finding for polymorphisms that nominally deviated from Hardy-Weinberg expectations in control samples.

Adjustments for multiple testing were lacking in most studies since only 9.5% of them made adjustments during statistical interpretation. Conversely, regression analyses to adjust for covariates were used in a high proportion of studies (72.4%). Likewise, the magnitude of effects has been appropriately reported in terms of hazard or odds ratios and their 95% CIs in most studies (75.9%). By contrast, adjustments for the underlying population stratification were nearly absent as part of the statistical toolbox of the studies (89.7%). As few as 2 studies (6.9%) supported the association in an independent validation sample [32,35]. Only 6 of 29 studies (20.7%) explored functional significance of variants associated with disease, either by evaluating the functionality of the associated polymorphism using gene reporter assays [26,37] or by its correlation with serum protein levels [22,27,43,46].

## Discussion

This quality assessment of genetic association studies with positive findings in susceptibility or outcome of ALI and ARDS identified a total of 29 articles and 16 genes. Due to our limited knowledge of the pathogenesis of these conditions and given that it is likely that many common genes and pathways contribute to the onset, course, or severity of these two forms of the same disease process, for the purpose of genetic susceptibility and outcome in this systematic review, we considered ALI and ARDS as a single entity. The top gene ontologies represented in current association studies fit within the major biological processes underlying ALI development on the basis of different microarray experiments among several studies using diverse animal models of the disease and cellular models of stretch-induced injury [51].

Overall, the paucity and quality of association data in ALI/ARDS call for more and better designed studies with larger sample sizes with unambiguous identification of the studied variants and procedures that allow monitoring of genotyping quality for a consistent replication and with better statistical analyses. Some of the reported associations, mostly in populations of European descent, have already set the bar high in the field with 'high-quality' studies, either with well-powered studies [36,41] or with a functional correlation of the associated polymorphism [43]. However, most of those association studies examining the functional effects of polymorphisms have reported the plasma levels of the gene product (protein) at one time point during the development or evolution of the disease process, so the role of those protein levels in the natural history of ALI or ARDS remains to be defined.

Additionally, positive association studies on ALI/ARDS have focused essentially on exploring genetic risk effects of candidate gene variants in European populations. Thus, future studies must try to fill this gap by extending the association analysis to other populations that might give us an overall picture of cosmopolitan and population-specific genetic risks. This also requires authors to give a more appropriate interpretation of results in light of power estimates since genetic effects are expected to be weak and sample sizes will rarely increase to the extent considered necessary [52]. The current evidence also encourages more replication studies, especially of those genes that have been positively associated in at least two studies [53]. A strong candidate would be the gene encoding the pro-inflammatory cytokine interleukin-6 (IL-6). Extensive cross-species gene expression pattern comparisons in experimental models of ALI have shown that IL-6 is highly upregulated [54] and at increased circulating concentrations in ALI patients [55]. However, undisputed evidence supporting the association of *IL-6 *gene variants with ALI/ARDS susceptibility or outcome is still lacking, even though positive results have been found in four studies. One of the major reasons is that the predicated association has been explored in a single polymorphism of the *IL-6 *gene (G/C at position -174 from the transcription start site). Association studies using a gene-wide coverage of common variation may reveal more robust patterns of variation associated with the disease [28,47]. In this sense, a (nearly) full coverage of common variation of the candidate gene in association studies of ALI is especially important since no association is yet definitive and our understanding of the functional elements of our genome is incomplete [56].

Classification and characterization of ALI/ARDS across reviewed studies were highly concordant. However, another face of the problem is that ALI/ARDS is still ill defined and the problem is further confounded by the diversity of etiological mechanisms such as sepsis, pneumonia, trauma, and massive transfusion that predispose patients to the condition. Furthermore, it has been recently shown that patients meeting current American-European Consensus Conference ARDS criteria may have highly variable levels of lung injury and outcomes [2]. We believe that the development of novel diagnostic tools to precisely characterize the ALI and ARDS phenotypes or the risk factors underlying disease development might result in associations that are more reproducible.

As a result of the progress of our understanding of this disease and the use of high-throughput methodologies [57], it is expected that robust well-replicated associations between genetic polymorphisms and ALI/ARDS susceptibility and outcome will become a reality in the near future. To reach this point, guidelines to report genotype data fulfilling minimum quality standards need to be implemented to improve our understanding of the genetic architecture of this disease. In addition, statistical methodologies such as multiple testing and population stratification adjustments, which to date have been almost completely absent in these studies, need to be routinely employed as well.

## Conclusion

Since all studied candidate genes await validation in independent studies using larger samples, the search for genetic variants determining susceptibility and outcome in ALI or ARDS still needs to grow and continue improving for the identification of true associations between genotype and clinical outcomes important in the care of ALI/ARDS patients. Integration of data across studies (for example, gene expression profiling, association studies, and proteomics) may reveal novel insights into ALI development which may allow us to identify cellular pathways specific to the disease. This knowledge will speed up the development of better and increasingly efficient tailored therapies for ALI/ARDS patients admitted to the intensive care unit.

## Key messages

• Current evidence suggests that acute lung injury (ALI) and its most severe form, the acute respiratory distress syndrome, are influenced by genetic factors.

• Association studies, the main tool for the exploration of common genetic variation underlying ALI, have thus far reported a total of 16 genes associated with ALI susceptibility and/or outcome.

• These genes are involved mainly in the response to external stimulus and cell signal transduction.

• More studies with improved designs, as well as replication of previous findings with larger sample sizes, are needed to definitely identify genetic factors predisposing patients to ALI.

## Abbreviations

ALI: acute lung injury; ARDS: acute respiratory distress syndrome; CI: confidence interval; FiO_2_: fraction of inspired oxygen; IL-6: interleukin-6; IQR: interquartile range; LD: linkage disequilibrium; NCBI: National Center for Biotechnology Information; PaO_2_: arterial partial pressure of oxygen.

## Competing interests

The authors declare that they have no competing interests.

## Authors' contributions

All authors contributed equally in the assessment design and the literature search and read and approved the final manuscript.

## References

[B1] Bernard GR, Artigas A, Brigham KL, Carlet J, Falke K, Hudson L, Lamy M, LeGall JR, Morris A, the Consensus Committee (1994). The American-European Consensus Conference on ARDS: definitions, mechanisms, relevant outcomes and clinical trial coordination. Am J Respir Crit Care Med.

[B2] Villar J, Pérez-Méndez L, López J, Belda J, Blanco J, Saralegui I, Suárez-Sipman F, López J, Lubillo S, Kacmarek RM, the HELP Network (2007). An early PEEP/FiO_2 _trial identifies different degrees of lung injury in patients with acute respiratory distress syndrome. Am J Respir Crit Care Med.

[B3] Rubenfeld GD, Caldwell E, Peabody E, Weaver J, Martin DP, Neff M, Stern EJ, Hudson LD (2005). Incidence and outcomes of acute lung injury. N Engl J Med.

[B4] Villar J, Maca-Meyer N, Pérez-Méndez L, Flores C (2004). Understanding genetic predisposition to sepsis. Crit Care.

[B5] Cobb JP, O'Keefe GE (2004). Injury research in the genomic era. Lancet.

[B6] Rahim NG, Harismendy O, Topol EJ, Frazer KA (2008). Genetic determinants of phenotypic diversity in humans. Genome Biology.

[B7] Sorensen TI, Nielsen GG, Andersen PK, Teasdale TW (1998). Genetic and environmental influences on premature death in adult adoptees. N Engl J Med.

[B8] Moss M, Mannino DM (2002). Race and gender differences in acute respiratory distress syndrome deaths in the United States: an analysis of multiple-cause mortality data (1979 – 1996). Crit Care Med.

[B9] Prows DR, Shertzer HG, Daly MJ, Sidman CL, Leikauf GD (1997). Genetic analysis of ozone-induced acute lung injury in sensitive and resistant strains of mice. Nat Genet.

[B10] Nonas SA, Moreno-Vinasco L, Ma SF, Jacobson JR, Desai AA, Dudek SM, Flores C, Hassoun PM, Sam L, Ye SQ, Moitra J, Barnard J, Grigoryev DN, Lussier YA, Garcia JG (2007). Use of consomic rats for genomic insights into ventilator-associated lung injury. Am J Physiol Lung Cell Mol Physiol.

[B11] Prows DR, Hafertepen AP, Winterberg AV, Gibbons WJ, Wesselkamper SC, Singer JB, Hill AE, Nadeau JH, Leikauf GD (2008). Reciprocal congenic lines of mice capture the aliq1 effect on acute lung injury survival time. Am J Respir Cell Mol Biol.

[B12] Cordell HJ, Clayton DG (2005). Genetic association studies. Lancet.

[B13] Risch N, Merikangas K (1996). The future of genetic studies of complex human diseases. Science.

[B14] The International HapMap Consortium (2007). A second generation human haplotype map of over 3.1 million SNPs. Nature.

[B15] Iles MM (2008). What can genome-wide association studies tell us about the genetics of common disease?. PLoS Genet.

[B16] Hattsersley AT, McCarthy MI (2005). What makes a good genetic association study?. Lancet.

[B17] Lohmueller KE, Pearce CL, Pike M, Lander ES, Hirschhorn JN (2003). Meta-analysis of genetic association studies supports a contribution of common variants to susceptibility to common disease. Nat Genet.

[B18] Neale BM, Sham PC (2004). The future of association studies: gene-based analysis and replication. Am J Hum Genet.

[B19] Ashburner M, Ball CA, Blake JA, Botstein D, Butler H, Cherry JM, Davis AP, Dolinski K, Dwight SS, Eppig JT, Harris MA, Hill DP, Issel-Tarver L, Kasarskis A, Lewis S, Matese JC, Richardson JE, Ringwald M, Rubin GM, Sherlock G (2000). Gene ontology: tool for the unification of biology. The Gene Ontology Consortium. Nature Genet.

[B20] NCI-NHGRI Working Group on Replication in Association Studies (2007). Replicating genotype-phenotype associations. Nature.

[B21] Lin Z, Pearson C, Chinchilli V, Pietschmann SM, Luo J, Pison U, Floros J (2000). Polymorphisms of human SP-A, SP-B, and SP-D genes: association of SP-B Thr131Ile with ARDS. Clin Genet.

[B22] Marshall RP, Webb S, Hill MR, Humphries SE, Laurent GJ (2002). Genetic polymorphisms associated with susceptibility and outcome in ARDS. Chest.

[B23] Marshall RP, Webb S, Bellingan GJ, Montgomery HE, Chaudhari B, McAnulty RJ, Humphries SE, Hill MR, Laurent GJ (2002). Angiotensin converting enzyme insertion/deletion polymorphism is associated with susceptibility and outcome in acute respiratory distress syndrome. Am J Respir Crit Care Med.

[B24] Quasney MW, Waterer GW, Dahmer MK, Kron GK, Zhang Q, Kessler LA, Wunderink RG (2004). Association between surfactant protein B +1580 polymorphism and the risk of respiratory failure in adults with community-acquired pneumonia. Crit Care Med.

[B25] Gong MN, Wei Z, Xu LL, Miller DP, Thompson BT, Christiani DC (2004). Polymorphism in the surfactant protein-B gene, and the risk of direct pulmonary injury and ARDS. Chest.

[B26] Ye SQ, Simon BA, Maloney JP, Zambelli-Weiner A, Gao L, Grant A, Easley RB, McVerry BJ, Tuder RM, Standiford T, Brower RG, Barnes KC, Garcia JG (2005). Pre-B-cell colony-enhancing factor as a potential novel biomarker in acute lung injury. Am J Respir Crit Care Med.

[B27] Ip WK, Chan KH, Law HK, Tso GH, Kong EK, Wong WH, To YF, Yung RW, Chow EY, Au KL, Chan EY, Lim W, Jensenius JC, Turner MW, Peiris JS, Lau YL (2005). Mannose-binding lectin in severe acute respiratory syndrome coronavirus infection. J Infect Dis.

[B28] Sutherland AM, Walley KR, Manocha S, Russell JA (2005). The association of interleukin 6 haplotype clades with mortality in critically ill adults. Arch Intern Med.

[B29] Nonas SA, Finigan JH, Gao L, Garcia JG (2005). Functional genomic insights into acute lung injury: role of ventilators and mechanical stress. Proc Am Thorac Soc.

[B30] Gong MN, Zhou W, Williams PL, Thompson BT, Pothier L, Boyce P, Christiani DC (2005). -308 and *TNFB *polymorphisms in acute respiratory distress syndrome. Eur Respir J.

[B31] Medford AR, Keen LJ, Bidwell JL, Millar AB (2005). Vascular endothelial growth factor gene polymorphism and acute respiratory distress syndrome. Thorax.

[B32] Gao L, Grant A, Halder I, Brower R, Sevransky J, Maloney JP, Moss M, Shanholtz C, Yates CR, Meduri GU, Shriver MD, Ingersoll R, Scott AF, Beaty TH, Moitra J, Ma SF, Ye SQ, Barnes KC, Garcia JG (2006). Novel polymorphisms in the myosin light chain kinase gene confer risk for acute lung injury. Am J Respir Cell Mol Biol.

[B33] Gong MN, Thompson BT, Williams PL, Zhou W, Wang MZ, Pothier L, Christiani DC (2006). Interleukin-10 polymorphism in position -1082 and acute respiratory distress syndrome. Eur Respir J.

[B34] Jerng JS, Yu CJ, Wang HC, Chen KY, Cheng SL, Yang PC (2006). Polymorphism of the angiotensin-converting enzyme gene affects the outcome of acute respiratory distress syndrome. Crit Care Med.

[B35] Gao L, Flores C, Fan-Ma S, Miller EJ, Moitra J, Moreno L, Wadgaonkar R, Simon B, Brower R, Sevransky J, Tuder RM, Maloney JP, Moss M, Shanholtz C, Yates CR, Meduri GU, Ye SQ, Barnes KC, Garcia JG (2007). Macrophage migration inhibitory factor in acute lung injury: expression, biomarker, and associations. Transl Res.

[B36] Bajwa EK, Yu CL, Gong MN, Thompson BT, Christiani DC (2007). Pre-B-cell colony-enhancing factor gene polymorphisms and risk of acute respiratory distress syndrome. Crit Care Med.

[B37] Marzec JM, Christie JD, Reddy SP, Jedlicka AE, Vuong H, Lanken PN, Aplenc R, Yamamoto T, Yamamoto M, Cho HY, Kleeberger SR (2007). Functional polymorphisms in the transcription factor NRF2 in humans increase the risk of acute lung injury. FASEB J.

[B38] Villar J, Pérez-Méndez L, Flores C, Maca-Meyer N, Espinosa E, Muriel A, Sangüesa R, Blanco J, Muros M, Kacmarek RM, the GRECIA and GEN-SEP Groups (2007). A CXCL2 polymorphism is associated with better outcomes in patients with severe sepsis. Crit Care Med.

[B39] Gong MN, Zhou W, Williams PL, Thompson BT, Pothier L, Christiani DC (2007). Polymorphisms in the *mannose binding lectin-2 *gene and acute respiratory distress syndrome. Crit Care Med.

[B40] Adamzik M, Frey U, Sixt S, Knemeyer L, Beiderlinden M, Peters J, Siffert W (2007). ACE I/D but not AGT (-6)A/G polymorphism is a risk factor for mortality in ARDS. Eur Respir J.

[B41] Zhai R, Zhou W, Gong MN, Thompson BT, Su L, Yu C, Kraft P, Christiani DC (2007). Inhibitor kappaB-alpha haplotype GTC is associated with susceptibility to acute respiratory distress syndrome in Caucasians. Crit Care Med.

[B42] Adamzik M, Frey UH, Rieman K, Sixt S, Beiderlinden M, Siffert W, Peters J (2007). Insertion/deletion polymorphism in the promoter of NFKB1 influences severity but not mortality of acute respiratory distress syndrome. Intensive Care Med.

[B43] Zhai R, Gong MN, Zhou W, Thompson TB, Kraft P, Su L, Christiani DC (2007). Genotypes and haplotypes of the VEGF gene are associated with higher mortality and lower VEGF plasma levels in patients with ARDS. Thorax.

[B44] Arcaroli J, Sankoff J, Liu N, Allison DB, Maloney J, Abraham E (2008). Association between urokinase haplotypes and outcome from infection-associated acute lung injury. Intensive Care Med.

[B45] Christie JD, Ma SF, Aplenc R, Li M, Lanken PN, Shah CV, Fuchs B, Albelda SM, Flores C, Garcia JG (2008). Variation in the MYLK gene is associated with development of acute lung injury after major trauma. Crit Care Med.

[B46] Garcia-Laorden MI, Sole-Violan J, Rodriguez de Castro F, Aspa J, Briones ML, Garcia-Saavedra A, Rajas O, Blanquer J, Caballero-Hidalgo A, Marcos-Ramos JA, Hernandez-Lopez J, Rodriguez-Gallego C (2008). Mannose-binding lectin and mannose-binding lectin-associated serine protease 2 in susceptibility, severity, and outcome of pneumonia in adults. J Allergy Clin Immunol.

[B47] Flores C, Ma SF, Maresso K, Wade M, Villar J, Garcia JGN (2008). An *IL6 *gene-wide haplotype is associated with susceptibility to acute lung injury. Transl Res.

[B48] Schroeder O, Schulte KM, Schroeder J, Ekkernkamp A, Laun RA (2008). The -1082 interleukin-10 polymorphism is associated with acute respiratory failure after major trauma: a prospective cohort study. Surgery.

[B49] Adamzik M, Frey UH, Riemann K, Sixt S, Lehmann N, Siffert W, Peters J (2008). Factor V Leiden mutation is associated with improved 30-day survival in patients with acute respiratory distress syndrome. Crit Care Med.

[B50] Yu W, Gwinn M, Clyne M, Yesupriya A, Khoury MJ (2008). A navigator for human genome epidemiology. Nat Genet.

[B51] Wurfel MM (2007). Microarray-based analysis of ventilator-induced lung injury. Proc Am Thorac Soc.

[B52] Zondervan KT, Cardon LR (2004). The complex interplay among factors that influence allelic association. Nat Rev Genet.

[B53] Villar J, Flores C, Pérez-Méndez L, Maca-Meyer N, Espinosa E, Blanco J, Sangüesa R, Muriel A, Tejera P, Muros M, Slutsky AS (2008). Angiotensin-converting enzyme insertion/deletion polymorphism is not associated with susceptibility and outcome in sepsis and acute respiratory distress syndrome. Intensive Care Med.

[B54] Grigoryev DN, Ma SF, Irizarry RA, Ye SQ, Quackenbush J, Garcia JG (2004). Orthologous gene-expression profiling in multi-species models: search for candidate genes. Genome Biology.

[B55] Meduri GU, Headley S, Kohler G, Stentz F, Tolley E, Umberger R, Leeper K (1995). Persistent elevation of inflammatory cytokines predicts a poor outcome in ARDS. Plasma IL-1 beta and IL-6 levels are consistent and efficient predictors of outcome over time. Chest.

[B56] ENCODE Project Consortium (2007). Identification and analysis of functional elements in 1% of the human genome by the ENCODE pilot project. Nature.

[B57] Fan JB, Chee MS, Gunderson KL (2006). Highly parallel genomic assays. Nat Rev Genet.

